# Acute pancreatitis and COVID-19: an integrative review of the literature

**DOI:** 10.1590/0100-6991e-20233559-en

**Published:** 2023-06-22

**Authors:** JULYANNE TEREZA CORDEIRO SILVA, OLIVAL CIRILO LUCENA DA FONSECA

**Affiliations:** 1 - Centro Universitário Maurício de Nassau - Recife - PE - Brasil; 2 - Hospital Universitário Oswaldo Cruz - Recife - PE - Brasil

**Keywords:** COVID-19, SARS-CoV-2, Pancreatitis, COVID-19, SARS-CoV-2, Pancreatite

## Abstract

The first cases of the COVID-19 disease were identified in late 2019 in China, but it didnt take long for it to become pandemic. At first, it was believed that it was restricted to respiratory symptoms only, until extrapulmonary manifestations were reported worldwide. Acute pancreatitis concomitant with the diagnosis of SARS-CoV-2 infection has been observed in some patients, in the absence of the most common etiologies described in the literature. It is postulated that the presence of the ECA-2 viral receptor in the pancreas is responsible for the direct cellular damage and that the hyperinflammatory state of COVID-19 favors the development of pancreatitis through an immune-mediated mechanism. This study aimed to analyze the correlation between acute pancreatitis and COVID-19 disease as a probable causality factor. An integrative literature review was carried out, including studies published between January 2020 and December 2022 that brought data on patients diagnosed with acute pancreatitis according to the revised Atlanta Classification with a confirmed diagnosis of COVID-19 in the same period. A total of thirty studies were reviewed. Demographic, clinical, laboratory and imaging aspects were analyzed and discussed. It is believed that SARS-CoV-2 was responsible for the development of acute pancreatitis in these patients, due to the absence of other precipitating risk factors, as well as the close temporal relationship between both. Attention should be given to gastrointestinal manifestations in patients affected by COVID-19.

## RESUMO

Os primeiros casos da doença COVID-19 foram identificados no final de 2019 na China, mas não foi necessário muito tempo para que se tornasse pandêmica. Acreditava-se, a princípio, que ela fosse restrita apenas a sintomas respiratórios, até que manifestações extrapulmonares fossem mundialmente relatadas. Quadros de pancreatite aguda concomitantes ao diagnóstico de infecção por SARS-CoV-2 vêm sendo observados em alguns pacientes, na ausência das etiologias mais comuns descritas na literatura. Postula-se que a presença do receptor viral ECA-2 no pâncreas seja responsável pelo dano celular direto e que o estado hiperinflamatório da COVID-19 favoreça o desenvolvimento da pancreatite por mecanismo imunomediado. Este estudo teve como objetivo analisar a correlação entre pancreatite aguda e a doença COVID-19 como um provável fator de causalidade. Realizou-se uma revisão integrativa da literatura, foram incluídos estudos publicados entre janeiro de 2020 e dezembro de 2022 que trouxessem dados acerca de pacientes diagnosticados com pancreatite aguda conforme a Classificação de Atlanta revisada com diagnóstico confirmado de COVID-19 no mesmo período. Um total de trinta estudos foram revisados. Aspectos demográficos, clínicos, laboratoriais e de imagem foram analisados e discutidos. Acredita-se que o SARS-CoV-2 foi o responsável pelo desenvolvimento de pancreatite aguda nestes pacientes, devido à ausência de demais fatores de risco precipitantes, bem como à estreita relação temporal entre ambos. Uma atenção deve ser dada às manifestações gastrointestinais em pacientes acometidos pela COVID-19.

## INTRODUCTION

In December 2019, Chinese authorities alerted the World Health Organization (WHO) to the appearance of cases of atypical pneumonia in the city of Wuhan^1^. After genetic sequencing, a new type of coronavirus was identified, defined as SARS-CoV-2, and the disease as COVID-19^1^ ^-^ ^3^. It was a single-stranded RNA virus, the seventh of the group of human coronaviruses to be identified, so designated for its high homology with SARS-CoV, responsible for outbreaks of Acute Respiratory Distress Syndrome (ARDS) between the years 2002 and 2003, also in China^1^ ^,^ ^3^. In January 2020, the WHO declared a Public Health Emergency of International Concern (PHEIC) and in March of the same year it was defined as a pandemic^1^. As of April 20, 2022, more than 6.2 million reported deaths have been attributed to COVID-19 worldwide^4^.

Initially, it was believed that it had a strictly respiratory character^3^. However, due to its rapid dissemination, reports of the most varied presentations of this disease have emerged, raising hypotheses of a multisystem involvement^5^. Gastrointestinal symptoms, in turn, may be present in up to half of the patients, with or without associated respiratory symptoms^5^. In the wake of the pandemic, it has already been possible to identify RNA from SARS-CoV-2 in stool samples from 48.1% of patients^6^, in peritoneal fluid^7^, and in the drainage of a pancreatic pseudocyst from a patient hospitalized shortly before for acute edematous pancreatitis of unknown origin^8^.

As well as the report of this latter patient, other authors also observed the occurrence of acute pancreatitis in patients infected with SARS-CoV-2 in the absence of known risk factors - cholelithiasis, alcoholism, hypertriglyceridemia, and others^9^. Particularly, infectious agents are responsible for about 10% of cases. Among the viral causes, there are coxsackie, hepatitis B, cytomegalovirus (CMV), human immunodeficiency virus (HIV), herpes simplex (HSV), mumps, and varicella-zoster^10^. It is postulated that the pancreatic involvement linked to COVID-19 occurs by two main mechanisms: 1) Direct cytotoxic injury: SARS-CoV-2 uses the angiotensin-converting enzyme 2 (ACE2) receptor to enter human cells; it has been discovered that this receptor is also expressed in the pancreas, both in exocrine glands and in islet cells, explaining the viral affinity for the organ^2,11 14^; and 2) Systemic hyperinflammation: the virus induces an exacerbated and unregulated immune response, known as a “cytokine storm”, thus resulting in multiple organ failure, including pancreatic^11^ ^,^ ^12^.

An international multicenter, prospective study observed that patients with acute pancreatitis and concomitant SARS-CoV-2 infection are not only subject to worse clinical outcomes (increased severity of pancreatitis, length of stay, and organ failure), but also display a significantly higher 30-day mortality rate^15^. 

## GOAL

Our study aims to analyze the correlation between acute pancreatitis and the disease COVID-19 as a probable causality factor, in the absence of other classically known etiologies.

## METHODS

This is an Integrative Literature Review conducted in six stages: identification of the problem, elaboration of a guiding question, literature search, evaluation and careful analysis of the data, and, finally, presentation of the review with its results and limitations^16^.

We performed the search in the PubMed database with the terms “Acute Pancreatitis” AND “adults” AND “COVID 19”, all available in MeSH and DeCS.

### Eligibility Criteria

Inclusion criteria were original, prospective or retrospective studies, reports, and case series, published between January 1^st^, 2020 and December 31^st^, 2022, in English, comprising cases and data about acute pancreatitis in adults associated with laboratory and/or radiologically confirmed COVID-19. The diagnosis of pancreatitis should comply with the revised Atlanta Classification^17^, with the presence of at least two of the following criteria: 1) abdominal pain consistent with acute pancreatitis (acute onset of intense and persistent epigastric pain, often radiating to the back); 2) lipase or amylase serum level three or more times the upper normal limit; and 3) imaging findings characteristic of acute pancreatitis on computed tomography (CT), magnetic resonance imaging (MRI), or ultrasonography (USG).

We excluded publications regarding cases of acute pancreatitis in children (<18 years), studies without full text available or pre-publications, and those that did not specify the diagnosis of acute pancreatitis based on the Atlanta Classification in the methods section. We also excluded reports of patients with known risk factors9 for pancreatitis, namely, biliary lithiasis, history of alcohol abuse, hypertriglyceridemia (>1000mg/dL), hypercalcemia, viral infections except SARS-CoV-2, patients undergoing recent invasive procedures such as ERCP (endoscopic retrograde cholangiopancreatography), autoimmune pancreatitis (elevated IgG4 levels), patients with recurrent acute pancreatitis or a family history that would raise suspicion of hereditary pancreatitis, medication abuse, and structural causes, such as pancreas divisum and nearby tumors or injuries.

Initially, we identified 94 studies. After reading the titles and abstracts, we excluded 49 of them, leaving 45 pre-selected for analysis of the full text. After full reading, we discarded 15, the final sample consisting of 30 studies chosen for data extraction and construction of the review.

## RESULTS

We reviewed 30 studies, including 24 four case reports^8,18 40^, two letters with case descriptions^41^ ^,^ ^42^, one prospective study with 316 patients in Turkey^43^, one Dutch cross-sectional study^44^, and two other retrospective studies, both from the United States^45^ ^,^ ^46^.

[Fig f1]

**Figure 1 f1:**
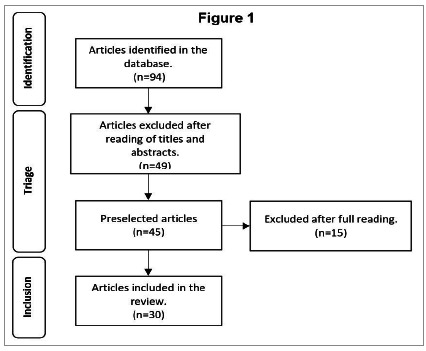
We analyzed the articles using the criteria of data reduction, display, and comparison.

Table 1 describes the general demographic profile (age and sex) of 31 patients from the case reports and letters, as well as symptomatology, pancreatic enzyme levels, presence or absence of characteristic findings of pancreatitis on imaging tests, and outcome (recovered - hospital discharge or description of clinical improvement - or death). Table 2 summarizes the main results of the larger studies and the methodology used.

**Table 1:** First author, demographics, clinical data, amylase and lipase levels, presence or absence of pancreatitis findings on imaging, and patient outcomes from case reports and letters.

1^st^ Author	Age and Sex	Constitutional and/or respiratory symptoms	Gastrointestinal manifestations	Serum lipase and amylase	Image findings suggestive of acute pancreatitis	Outcome
Aloysius et al.^18^	36/F	Fever, cough, dyspnea	Epigastric abdominal pain and tenderness radiating to the back, nausea, vomiting, diarrhea	L: 627U/L A: 325U/L	No abnormalities	Recovered
Sharma et al.^41^	25/F	Fever, headache, chills, odynophagia	Excruciating epigastric abdominal pain and tenderness radiating to the back, vomiting	L: 11920,7U/L A: 1814,6U/L	Yes	Recovered
Almutairi et al.^19^	71/M	Fever, cough, dyspnea	Abdominal pain with epigastric tenderness radiating to the right upper quadrant	L: 1023U/L A: 544U/L	Yes	NR
Nizamic et al.^20^	49/F	No respiratory symptoms	Abdominal pain, diarrhea, limited oral intake	L: 2864U/L A: NR	Yes	Recovered
AlHarmi et al.^21^	52/F	Fever, cough, dyspnea	Burning epigastric and right upper quadrant abdominal pain radiating to the back, worsened by oral ingestion, nausea, and vomiting	L: NR A: 47U/L	Yes	Recovered
1^st^ Author	Age and Sex	Constitutional and/or respiratory symptoms	Gastrointestinal manifestations	Serum lipase and amylase	Image findings suggestive of acute pancreatitis	Outcome
Tadkal et al.^22^	42/M	Fever, headache	Abdominal pain	L: 119U/L A: 136U/L	Yes	Recovered
	71/M	Fever, cough, dyspnea	Abdominal pain and distention	L: 143U/L A: 93U/L	Yes	Recovered
	63/M	Fever, cough, dyspnea	Abdominal pain, nausea, and vomiting	L: 178U/L A: 139U/L	Yes	Recovered
	36/M	Fever, cough	Abdominal pain with tenderness in the umbilical region, vomiting	L: 185U/L A: 204U/L	Yes	Recovered
	28/M	Febre, tosse	Dor abdominal com sensibilidade em região umbilical, vômitos	L: 2851U/L A: 834U/L	Yes	Óbito
Arbati et al.^23^	28/M	Fever, cough, dyspnea, myalgia	Severe epigastric abdominal pain radiating to the back, bloating, nausea, and vomiting	L: 758U/L A: 1273U/L	Yes	Recovered
Cheung et al.^24^	38/M	Fever	Severe epigastric abdominal pain radiating to the back, nausea, and vomiting	L: 20320ukat/L A: U/L	Yes	Recovered
Ferreira et al.^25^	35/M	Dyspnea	Sharp epigastric abdominal pain and tenderness radiating to the back, nausea, and vomiting	L: NR A: 1669U/L	Yes	Recovered
Simou et al.^26^	67/ NR	Fever, dyspnea, myalgia, arthralgia	No characteristic gastrointestinal symptoms	L: 576U/L A: NR	Yes	Death
Hatch-Vallier et al.^27^	39/F	Fever, cough, ageusia, anosmia	Epigastric abdominal pain and tenderness, nausea	L: 43U/L A: NR	Yes	Recovered
Kumaran et al.^28^	67/F	No respiratory symptoms	Epigastric abdominal pain, nausea and vomiting, diarrhea	L: NR A: 1483U/L	Yes	Recovered
Jeelani et al.^29^	24/M	Fever, cough, dyspnea	Epigastric abdominal pain and tenderness, nausea and vomiting, diarrhea	L: 2025U/L A: NR	Yes	Recovered
Eldaly et al.^30^	44/M	No respiratory symptoms	Severe epigastric abdominal pain and tenderness radiating to the back, vomiting	L: 286U/L A: 773U/L	Yes	Recovered
Narang et al.^31^	20/F	Fever, cough, dyspnea, myalgia	Acute epigastric abdominal pain radiating to the back, nausea, and vomiting	L: 916U/L A: 396U/L	Yes	Recovered
Mitrovic et al.^32^	33/M	No respiratory symptoms	Epigastric abdominal pain radiating to the back, nausea, and vomiting	L: 1082U/L A: 1426U/L	Yes	Óbito
Hadi et al.^33^	47/F	Fever, headache, dyspnea	No characteristic gastrointestinal symptoms	L: NR A: 1500U/L	Yes	NR
1^st^ Author	Age and Sex	Constitutional and/or respiratory symptoms	Gastrointestinal manifestations	Serum lipase and amylase	Image findings suggestive of acute pancreatitis	Outcome
	68/F	fever, fatigue	Epigastric and periumbilical abdominal pain and tenderness, abdominal distention, vomiting, diarrhea	L: NR A: 934U/L	NR	NR
Brikman et al.^34^	61/M	Fever, cough, dyspnea	Sudden onset diffuse abdominal pain and tenderness, anorexia	L: 203U/L A: 142U/L	Yes	Recovered
Fernandes et al.^42^	36/F	Fever, dyspnea, headache	Upper abdominal pain	L: 640U/L A: 710U/L	Yes	Recovered
Karimzadeh et al.^35^	65/F	Myalgia, chills; did not have respiratory symptoms on admission	Upper abdominal pain and tenderness, nausea	L: 283U/L A: 192U/L	No abnormalities	Recovered
Ghosh et al.^36^	63/M	Fever, cough, dyspnea	No characteristic gastrointestinal symptoms	L: 412U/L A: 58U/L	Yes	Recovered
Ibrahim et al^37^	33/M	Dyspnea	Abdominal pain with epigastric and right upper quadrant tenderness	L: >1200U/L A: 390U/L	Yes	Recovered
Bokhari et al.^38^	32/M	Fever, cough, myalgia, chills, odynophagia	Severe epigastric abdominal pain radiating to the back, nausea and vomiting, diarrhea	L: 721U/L A: 672U/L	Yes	Recovered
Kurihara et al.^39^	55/NR	Fever, cough	Description of abdominal pain not possible because of sedation	L: 263U/L A: 252U/L	Yes	Recovered
Abraham et al.^40^	61/F	Fever, dyspnea	Periumbilical abdominal pain, vomiting	L: 904U/L A: NR	NR	Recovered
Schepis et al.^8^	67/F	Fever	Epigastric and mesogastric abdominal pain and tenderness, abdominal distention, vomiting	L: 900U/L* A: NR	Yes	NR

F: Female; M: Male; NR: not reported. *Level of lipase in the fluid of a pancreatic pseudocyst.

**Table 2:** First author, objective, methods, and main results obtained by unpublished prospective and retrospective studies.

1^st^ Author	Goal	Methodology	Main results
Akarsu et al.^43^	To investigate pancreatic damage caused by SARS-CoV-2 and the effects of developing acute pancreatitis on the progression of COVID-19.	Data from 316 patients admitted to the institution between March 25, 2020 and April 25, 2020 with a diagnosis of COVID-19 pneumonia were prospectively evaluated. Diagnosis of COVID-19 was via RT-PCR and CT findings. The diagnosis of pancreatitis was according to the revised Atlanta criteria. Patients were categorized into three levels of pneumonia severity (mild, severe, critical). Demographics, pancreatitis rate, biochemical, and radiological parameters of each group were analyzed. Patients were divided into two groups and the results were compared: COVID-19 patients with acute pancreatitis (Group P) and without acute pancreatitis (Group C).	Acute pancreatitis was detected in 12.6% of the 316 patients. The mean age for those with acute pancreatitis was 55. There was a positive correlation between advanced age and mortality (p=0.0003). Males constituted 59.1% of the patients. Sex did not make a significant difference in terms of mortality (p=0.3999) and development of pancreatitis (p=0.4192). Gastrointestinal symptoms such as abdominal pain, nausea, vomiting, and diarrhea were observed in 87.5% of patients who had pancreatitis. There were 50 patients (15.8%) in mild condition, 189 patients (59.8%) in severe condition and 77 patients (24.3%) in critical condition. There was no acute pancreatitis in mild patients; 7.9% of patients in severe condition and 32.5% of patients in critical condition had acute pancreatitis. There was a positive correlation between the severity of pneumonia and pancreatitis, and the rate of pancreatitis increased with pneumonia severity (p<0.0001). Hospitalization and mortality rates were higher in patients with COVID-19 accompanied by acute pancreatitis (p=0.0038 and p<0.0001, respectively).
Kumar et al.^45^	To describe the epidemiology, clinical course, and outcome of patients hospitalized with COVID-19 and acute pancreatitis.	Retrospective analysis of the data record of all adult patients (>18 years old) who were admitted with a diagnosis of COVID-19 and acute pancreatitis (at the same encounter) from February 1 to June 30, 2020, at one of five Partners Hospitals Healthcare Network. COVID-19 disease was confirmed by RT-PCR. The diagnosis of acute pancreatitis was made according to the revised Atlanta Classification. Detailed data including demographics, symptoms, respiratory failure, shock, ICU transfer, mechanical ventilation, laboratory abnormalities, and imaging findings were recorded.	Of 985 patients screened, 17 were eligible for the study, nine (52.9%) were hospitalized primarily with ARDS associated with coronavirus disease, requiring intubation and mechanical ventilation. These patients developed acute pancreatitis after a median of 22.5 days (range 13 76) from the onset of COVID-19 symptoms. In contrast, eight patients had typical symptoms and were diagnosed with acute pancreatitis on admission. Of eight, three (37.5%) developed respiratory and constitutional symptoms of COVID-19, one (12.5%) before the diagnosis of acute pancreatitis, while two patients (25%) developed fever and cough after three days of hospitalization. Patients admitted primarily with severe COVID-19 were younger (mean age 57 vs. 63 years), female (55.6% vs. 25%), Hispanic ethnicity (55.6% vs. 25%), and obese (88.9% vs. 37.5%). Of the 17 patients, the median lipase peak among mechanically ventilated ones was higher (661 vs. 236U/L). One patient in each group did not have lipase elevation but had clinical courses and CT images characteristic of acute pancreatitis. In addition, levels of other markers used to monitor COVID-19 disease, including CRP, ferritin, lactate dehydrogenase, and D-dimer were higher among patients who subsequently developed acute pancreatitis. Although the triglyceride spike preceded the diagnosis of acute pancreatitis among these patients, triglyceride levels rapidly declined upon discontinuation of propofol and/or tube feeding. Among nine patients who subsequently developed acute pancreatitis, five (55.6%) experienced thromboembolic complications compared with one patient (12.5%) who presented with acute pancreatitis upon admission. Among patients who subsequently developed acute pancreatitis, three died of ARDS and multisystem organ failure, resulting in an in-hospital mortality rate of 33.3%. Patients hospitalized for acute pancreatitis had an in-hospital mortality rate of 12.5%.
Bulthuis et al.^44^	To investigate the incidence, severity, and clinical impact of acute pancreatitis in patients with COVID-19.	Cross-sectional study of a prospective, observational cohort of all COVID-19 patients admitted to two Dutch teaching hospitals between March 4, 2020 and May 26, 2020. COVID-19 was defined as a positive result on high-report sequencing yield or via RT-PCR and/or characteristic chest CT findings. Acute pancreatitis was defined according to the revised Atlanta Classification. The primary outcome was acute pancreatitis potentially related to COVID-19 infection. A potential relationship with COVID-19 was defined as the absence of a clear etiology of acute pancreatitis. Secondary outcomes included the development of pancreatic necrosis, organ failure, and the clinical impact of acute pancreatitis.	Among 433 patients with COVID-19, five (1.2%) had potentially related acute pancreatitis according to the revised Atlanta Classification. The median age of these five patients was 60 years (47 71). Four patients (80%) were male. These five patients were suffering from severe COVID-19 infection. All suffered organ failure due to COVID-19: three (60%) suffered from respiratory failure, two (40%) renal, and three (60%) cardiovascular. Four patients were admitted to the ICU for an average of 14 days (12 14). All patients with acute pancreatitis underwent contrast-enhanced abdominal CT. The median length of stay was 21 days (20 35). No additional interventions related to acute pancreatitis were performed. Three patients (60%) died.
Gubatan et al.^46^	To report the point prevalence, risk factors, and outcomes of hospitalized patients with COVID-19 presenting with acute pancreatitis and compare the pancreatitis outcomes in patients without COVID-19.	Retrospective, observational cohort study of patients aged 18 years and older admitted to 12 hospitals within the Northwell Health System from March 1, 2020 to June 1, 2020, in New York City. Patients were identified as having acute pancreatitis if they fulfilled all three criteria of the revised Atlanta Classification. Those with acute pancreatitis and COVID 19 were compared to a group of patients with acute pancreatitis but without COVID 19. Patients’ charts were manually reviewed not only to confirm the diagnosis of pancreatitis, but also to determine its etiology. The primary outcomes of mortality, length of stay, need for mechanical ventilation, and development of pancreatic necrosis was compared between the two groups.	During the study period, 48,012 patients were hospitalized and of them 11,883 (24.75%) were positive for COVID-19 at admission. A total of 189 had a diagnosis of pancreatitis (point prevalence 0.39%) and 32 of the 189 (17%) were positive for COVID 19, yielding a point prevalence of 0.27% for pancreatitis among hospitalized patients with COVID 19. There was a higher proportion of Black and Hispanic patients with pancreatitis in the COVID positive group compared with the COVID negative group. Among the group of negative COVID-19 patients, gallstone and alcohol etiologies were the most common, with 34% and 37%, respectively, similar to the general population. However, among patients with COVID-19, these etiologies accounted for only 16% and 6% of cases, respectively. Instead, idiopathic pancreatitis was the most common etiology in this group - 69%, compared with 21% in patients who were COVID negative (p<0.0001). Patients with pancreatitis who were also COVID positive were more likely to require mechanical ventilation and had longer hospital stays compared with patients with pancreatitis without COVID 19 (OR 5.65, p=0.01 and OR 3.22, p=0.009, respectively).

The mean age of the 31 patients described in the 24 reports and two letters was 46.97 years, two of them did not specify the patients sexes^26^ ^,^ ^39^, in the others most were male (16/29). Past medical history mostly included systemic arterial hypertension^19^ ^,^ ^21^ ^,^ ^22^ ^,^ ^28^ ^,^ ^32^ ^,^ ^33^ ^,^ ^35^ ^,^ ^40^, obesity^18^ ^,^ ^21^ ^,^ ^25^ ^,^ ^26^ ^,^ ^31^, diabetes mellitus^19^ ^,^ ^21^ ^,^ ^22^ ^,^ ^26^ ^,^ ^36^,and kidney disease chronic^19^ ^,^ ^20^ ^,^ ^22^ ^,^ ^32^ ^,^ ^40^. Some patients did not have a significant medical history^24^ ^,^ ^27^ ^,^ ^29^ ^,^ ^30^ ^,^ ^33^ ^,^ ^34^ ^,^ ^38^ ^,^ ^39^ ^,^ ^41^. One had undergone laparotomy one year before for intestinal resection due to superior mesenteric artery stenosis^28^. Another was in the 33^rd^ week of her first child pregnancy child^31^.

The diagnosis of SARS-CoV-2 infection was primarily confirmed by RT PCR (real-time polymerase chain reaction) with nasopharyngeal swab samples^18 26,28,30,31,34-40,42^; two patients underwent rapid tests^27^ ^,^ ^32^ ; two studies did not specify which diagnostic test was performed^33^ ^,^ ^41^. Chest radiological findings were limited to bilateral, peripheral, ground-glass opacities^8,18,21 23,25,26,31 35,37,42^.

Twelve patients had constitutional and/or respiratory and gastrointestinal symptoms upon admission, diagnosed as acute pancreatitis and COVID-19 during the same hospitalization^18^ ^,^ ^22^ ^,^ ^23^ ^,^ ^25^ ^,^ ^27^ ^,^ ^33^ ^,^ ^37^ ^,^ ^40^. Six were admitted with constitutional and/or respiratory symptoms and developed acute pancreatitis during hospitalization^21^ ^,^ ^26^ ^,^ ^31^ ^,^ ^33^ ^,^ ^34^ ^,^ ^39^. Among these, one later reported that the abdominal pain had been present for at least a week, but was initially treated as gastritis^21^. One patient did not manifest gastrointestinal symptoms and therefore his diagnosis was given by altered laboratory and imaging exams^26^ and in another there was no record of abdominal pain due to sedation^39^.

Five patients were initially diagnosed and treated for COVID-19 and later returned to the health service with gastrointestinal symptoms, receiving a diagnosis of pancreatitis^19^ ^,^ ^24^ ^,^ ^29^ ^,^ ^38^ ^,^ ^41^. One of them returned to the emergency room two days after discharge complaining of abdominal pain and was readmitted for acute gastritis; only later was acute pancreatitis investigated19. Two of these patients had been diagnosed with COVID-19 two^29^ and one^24^ week before, but on this second admission the test was still positive. The latter^24^ had already been diagnosed with “acute pancreatitis of uncertain etiology” on their first admission but were discharged and instructed to isolate themselves at home.

One specific patient was also diagnosed with SARS-CoV-2 infection in a first hospitalization, but developed drowsiness and was readmitted two days after discharge; the diagnosis of acute pancreatitis, in turn, was based on altered laboratory and imaging exams, since there were no reports of typical gastrointestinal symptoms^36^.

Gastrointestinal symptoms were the only manifestation of COVID-19 in six patients, despite the test positivity^8^ ^,^ ^20^ ^,^ ^28^ ^,^ ^30^ ^,^ ^32^ ^,^ ^35^. Only one letter did not bring information of this nature^42^.

The diagnosis of acute pancreatitis in all 30 included studies followed the revised Atlanta Classification^17^. Particularly, the study by Gubatan et al. required the presence of all three classification criteria, instead of a minimum of two of them^46^.

CT was the imaging exam of choice to visualize pancreatic changes. Some underwent MRI, as in the case of the pregnant patient^31^. The main findings refer to pancreatic and peripancreatic inflammatory alterations^19^ ^,^ ^24^ ^,^ ^27^ ^,^ ^29^ ^,^ ^31^ ^,^ ^38^, such as enlarged pancreas^22^ ^,^ ^25^ ^,^ ^26^ ^,^ ^30^ ^,^ ^32^ ^,^ ^33^ ^,^ ^36^ ^,^ ^38^ ^,^ ^39^ ^,^ ^41^, with heterogeneous density^23^ ^,^ ^34^, and even areas with no enhancement, indicative of necrotizing pancreatitis^22^ ^,^ ^28^ ^,^ ^32^ ^,^ ^36^, in addition to edema and peripancreatic fat densification^20^ ^-^ ^23^ ^,^ ^25^ ^,^ ^26^ ^,^ ^28^ ^,^ ^29^ ^,^ ^32^ ^,^ ^34^ ^,^ ^37^ ^,^ ^39^ ^,^ ^41^ ^,^ ^42^, with free fluid^22^ ^,^ ^23^ ^,^ ^25^. A CT scan of one patient revealed acute necrotic collections involving the head of the pancreas and the pancreaticoduodenal sulcus, and a 16mm central enhancement component seen within this formation, showing contrast extravasation, indicating a pseudoaneurysm of the pancreaticoduodenal artery^32^.

A pancreatic pseudocyst measuring 16cm x 8cm x 12cm was also identified, causing partial obstruction of the gastric outlet of one patient who had a history of recent hospitalization for acute edematous pancreatitis of unknown origin. Analysis of the fluid showed no bacterial growth, normal carcinoembryonic antigen (CEA) levels, elevated amylase levels (900U/L), and a SARS-CoV-2 RT-PCR positive for all three surveyed SARS-CoV-2 target genes^8^.

The search for precipitating risk factors for pancreatitis followed the tripod of clinical history, laboratory tests, and imaging. It is worth mentioning that not all reports brought all data. There was no history of smoking^18^ ^,^ ^25^ ^-^ ^28^ ^,^ ^30^ ^,^ ^32^ ^,^ ^33^ nor alcoholism or alcohol intake was minimal^18^ ^-^ ^28^ ^,^ ^30^ ^-^ ^34^ ^,^ ^36^ ^,^ ^38^ ^,^ ^41^ ^,^ ^42^. Drug abuse was also denied, except for continuous use to treat comorbidities and current health conditions^22^ ^-^ ^24^ ^,^ ^27^ ^,^ ^30^ ^-^ ^32^ ^,^ ^34^ ^,^ ^41^ ^,^ ^42^. Some patients did not report similar previous episodes or family history that would suggest a chronic nature or genetic predisposition^18^ ^,^ ^21^ ^,^ ^25^ ^-^ ^27^ ^,^ ^30^ ^,^ ^34^ ^,^ ^40^, there was no abdominal trauma^21^ ^,^ ^24^ ^,^ ^41^ ^,^ ^42^, recent abdominal surgery, or submittance to invasive procedures^21^ ^,^ ^24^ ^,^ ^25^ ^,^ ^41^.

During laboratory investigation, the most common alterations were leukocytosis^19^ ^,^ ^21^ ^-^ ^25^ ^,^ ^27^ ^-^ ^32^ ^,^ ^34^ ^,^ ^37^ ^,^ ^38^, elevated CRP values^18^ ^,^ ^19^ ^,^ ^22^ ^,^ ^25^ ^-^ ^28^ ^,^ ^30^ ^,^ ^32^ ^,^ ^33^ ^,^ ^36^ ^,^ ^38^ ^-^ ^41^, lactate dehydrogenase^20^ ^,^ ^27^ ^,^ ^28^ ^,^ ^38^, D-dimer^19^ ^,^ ^20^ ^,^ ^22^ ^,^ ^27^ ^,^ ^31^ ^,^ ^39^ ^,^ ^40^, IL-6^22^ ^,^ ^41^, ESR^40^ ^,^ ^41^, and ferritin^26^ ^,^ ^40^.

The triglyceride level was adequate^18^ ^,^ ^22^ ^-^ ^24^ ^,^ ^29^ ^,^ ^30^ ^,^ ^33^ ^,^ ^35^ ^,^ ^36^ ^,^ ^38^ ^,^ ^41^ or slightly elevated^19^ ^-^ ^21^ ^,^ ^25^ ^-^ ^28^ ^,^ ^34^ ^,^ ^39^ in most patients, but none above >1000mg/dL. Hypercalcemia was also not identified^19^ ^,^ ^21^ ^,^ ^22^ ^,^ ^24^ ^-^ ^29^ ^,^ ^33^ ^-^ ^40^. Bilirubin parameters were also within normal limits^18^ ^,^ ^21^ ^,^ ^23^ ^,^ ^24^ ^,^ ^27^ ^,^ ^30^ ^,^ ^34^ ^,^ ^38^ ^,^ ^39^ ^,^ ^41^, except in two patients^25^ ^,^ ^28^, as well as alkaline phosphatase^18^ ^,^ ^23^ ^,^ ^29^ ^,^ ^34^ ^,^ ^38^ ^,^ ^41^. Liver transaminase values varied, but most remained normal or slightly increased^18^ ^,^ ^22^ ^,^ ^23^ ^,^ ^26^ ^,^ ^27^ ^,^ ^29^ ^,^ ^31^ ^,^ ^34^ ^,^ ^35^ ^,^ ^38^ ^,^ ^39^ ^,^ ^41^. There were three reports of normal IgG4 values when investigating autoimmune pancreatitis^28^ ^,^ ^29^ ^,^ ^31^.

Four patients underwent further viral serological tests, all of which were negative. The first performed tests for HAV, HBV, HCV, HDV, HEV, HSV, VZV, EBV, CMV, and HIV^26^; the second cited only “tests for hepatitis”^28^; the third mentioned serologies for HBV, HCV, and HIV30; and the fourth, influenza A and B^35^.

The search for a biliary etiology was described in the vast majority of reports, none showing evidence of cholelithiasis or changes in the biliary tract on imaging tests - ultrasound and abdominal tomography^18^ ^,^ ^19^ ^,^ ^22^ ^-^ ^25^ ^,^ ^27^ ^-^ ^30^ ^,^ ^32^ ^-^ ^34^ ^,^ ^37^ ^-^ ^39^ ^,^ ^41^ ^,^ ^42^. Three patients had undergone cholecystectomy in the past^21^ ^,^ ^26^ ^,^ ^31^, the other reports not specifically mentioning radiological investigation for cholelithiasis, but generically denying its presence^20^ ^,^ ^36^ ^,^ ^40^. The patient who developed a pseudocyst had his previous pancreatitis classified as of “unknown origin”, thus, we assume that the biliary cause was investigated and ruled out^8^.

The management of these patients was conservative. Supportive care included pain control, antiemetics, bowel rest, and intravenous fluid resuscitation; empirical antibiotic therapy was also performed; a gradual return to oral feeding was advocated as tolerated^18^ ^,^ ^19^ ^,^ ^21^ ^-^ ^30^ ^,^ ^33^ ^-^ ^42^.

On the sixth day of hospitalization, the pregnant patient had premature rupture of membranes and subsequently evolved into active labor. After delivery, she returned to the ICU and progressed with an improvement in epigastric discomfort and respiratory function, with a reduction in amylase and lipase levels. She was transferred to the obstetric unit in room air on the second postpartum day and was discharged from the hospital on the third^31^.

The patient in whom the pancreaticoduodenal artery pseudoaneurysm was visualized was initially treated conservatively. However, due to his clinical deterioration, endovascular embolization was proposed as the best therapeutic option. He would be referred to another hospital to undergo the procedure, when massive rectal bleeding suddenly occurred. Despite all efforts to stabilize him, he died of hypovolemic shock. A tomography performed during resuscitation revealed active hemorrhage due to complete rupture of the pseudoaneurysm, associated with a more extensive area of pancreatic necrosis than previously visualized^32^.

The case of the pancreatic pseudocyst was solved by transgastric drainage guided by endoscopic ultrasound^8^.

Several patients required ventilatory support and/or ICU care at some point during their stay^18^ ^,^ ^19^ ^,^ ^22^ ^,^ ^23^ ^,^ ^25^ ^,^ ^26^ ^,^ ^28^ ^,^ ^31^ ^,^ ^33^ ^-^ ^37^ ^,^ ^39^ ^,^ ^41^. One of them was treated with veno-venous Extracorporeal Membrane Oxygenation (ECMO)^39^.

The mortality rate among the 31 patients was around 10%. It is worth mentioning that the clinical outcomes of four patients were not informed^8^ ^,^ ^19^ ^,^ ^33^.

## DISCUSSION

It was possible to summarize the currently available data on the development of acute pancreatitis concomitant with COVID-19, describing demographics, clinical presentation, laboratory and imaging findings, management, and outcomes. Although there is no demonstrably clear evidence that SARS-CoV-2 infection is responsible for acute pancreatitis, the results obtained from this review corroborate this hypothesis due to the absence of the most common risk factors, as well as the close temporal relationship between both.

The general incidence of acute pancreatitis is 110 to 140 per 100,000 inhabitants^9^. Most of these patients have mild acute pancreatitis, which is self-limiting and usually resolves within a week. About 20% of patients develop moderate or severe conditions, with a mortality rate of 20 to 40%^47^. Few studies informed patients’ severity stratification; therefore, we did not find it appropriate to include it in the results.

The etiologies responsible for acute pancreatitis include gallstones and alcohol mainly, in addition to hypertriglyceridemia, hypercalcemia, iatrogenic causes, viral, bacterial, and fungal infections, hereditary, autoimmune, drugs, and anatomical-structural factors^9^ ^,^ ^10^ ^,^ ^47^. The possibility of viral pancreatitis is already known and well established in the literature, with hepatotropic viruses, Coxsackie, CMV, HIV, HSV, mumps, varicella-zoster virus, and others^10^. One of the included studies, carried out by Gubatan et al., compared a group of patients with “pancreatitis without COVID-19” to another with “pancreatitis and COVID-19”, finding that the biliary and alcoholic etiology were the most common in the first, similar to the general population, while “idiopathic pancreatitis” curiously was present in 69% of cases in the second group^46^.

So far, pancreatic injury associated with COVID-19 has been postulated to come from two main pathophysiological mechanisms: direct cytopathic effect and dysregulated immune response.

SARS-CoV-2 uses the Angiotensin-Converting Enzyme-2 (ACE-2) receptor to enter human cells^3^. This receptor is present in lung type 2 alveolar cells, intestinal enterocytes, vascular endothelium, heart, kidneys, adrenals, pancreas, skeletal muscle, and adipose tissues^13^. The study by Liu et al. observed that the ACE-2 receptor was expressed both in the exocrine glands and in the pancreatic islets, and that its expression in this organ may be higher than in the lungs^14^. This would explain the viral tropism for the pancreas and the mechanism of direct injury.

Another pathophysiological explanation for the development of acute pancreatitis, which does not exclude the previous one, refers to the hyperinflammatory state of COVID-19 patients. The systemic and exacerbated inflammatory response triggered by SARS-CoV-2 results in the so-called cytokine storm, with uncontrolled production of pro-inflammatory cytokines, such as IL-6, TNF-α, and chemokines, leading to multiple organ failure, including pancreatic^11^ ^,^ ^12^ ^,^ ^48^.

We included studies that specified the diagnosis of acute pancreatitis according to the Atlanta Classification^17^. Particularly, the study by Gubatan et al. only considered acute pancreatitis in those patients who fulfilled the three criteria, which may have underestimated the rate of pancreatitis in their sample, since the diagnosis requires a minimum of two of them. The prevalence of pancreatitis seen by them among patients hospitalized with COVID-19 was 0.27%^46^.

The first criterion refers to the presence of characteristic abdominal pain^17^. It is already known that gastrointestinal symptoms can arise in the context of COVID-19^6^. Pan et al. observed that 103 hospitalized patients (50.5%) had one or more digestive symptoms. Among them, 97 developed respiratory symptoms associated with digestive symptoms and six had only digestive symptoms in the absence of respiratory symptoms. They also found that as disease severity increases, digestive symptoms become more pronounced^5^. 

Abdominal pain was present in most reports, not being detailed by some of them, unfortunately, regarding location, intensity, and irradiation. Akarsu et al. observed abdominal pain, nausea, vomiting, and diarrhea in almost 90% of the patients with pancreatitis^43^. This highlights the importance for health teams to be alert to the gastrointestinal manifestations of this disease, as they may be indicative of a multisystem involvement, especially pancreatic, as well as to recognize that one of the probable diagnoses of isolated acute abdominal conditions may be an infection by SARS -CoV-2, even in the absence of associated respiratory symptoms.

However, typical abdominal pain may not be present or be much less clear, as in the case of patients under sedation^39^. Therefore, laboratory and radiological investigations of pancreatitis become imperative.

The second diagnostic criterion consists of serum lipase or amylase activity at least three times greater than the upper normality limit^17^. This was a criterion present in most patients. Lipase has a slightly higher sensitivity for detecting acute pancreatitis; its elevation occurs earlier and lasts longer than amylase’s^49^. Normal serum levels of lipase are uncommon in clinical practice. Nonetheless, the patient reported by Hatch-Vallier et al., for example, had a normal level of lipase and her diagnosis was achieved by the presentation of characteristic epigastric pain and radiological findings^27^. In the study by Pandanaboyana et al., 87% of patients who were positive for COVID-19 within 14 days of admission had hyperamylasemia and abdominal pain suggestive of concomitant SARS-CoV-2 infection and acute pancreatitis^15^.

On the other hand, the isolated increase of pancreatic enzymes during COVID-19 is quite questionable and has been discussed by the scientific community. Wang et al. observed a 17% incidence of pancreatic injury among 52 patients infected with SARS-CoV-2; they defined “pancreatic injury” as any abnormality in the levels of amylase or lipase50. However, these enzymes can be secreted by organs other than the pancreas^19^ ^,^ ^51^. This increase, from an individual perspective, could also be explained by factors other than the probable pancreatic damage in patients with COVID-19, such as acidosis, renal failure, and diabetes^51^. A clear example was seen in a cohort of 110 patients diagnosed with COVID-19, in 24.5% of them displaying increased levels of amylase and 16.4%, increased levels of lipase, but only one patient had levels more than three times the upper limit and none developed clinical or radiological signs of acute pancreatitis^52^.

Therefore, we emphasize that this laboratory alteration should be considered and serve as a warning during pneumonia, but is does not, by itself, define acute pancreatitis. For this, the other criteria must be evaluated.

The third and last is related to findings consistent with acute pancreatitis on contrast-enhanced computed tomography (more common), magnetic resonance imaging, or ultrasonography^17^. It is true that pancreatitis can be diagnosed in approximately 80% of patients based only on the presence of classic abdominal pain and elevated pancreatic enzymes. However, CT is a useful complement to confirm the diagnosis in the absence of one of the other criteria and for the differential diagnosis with other intra-abdominal conditions that may mimic it^9^ ^,^ ^49^.

Acute pancreatitis can be subdivided into interstitial edematous and necrotizing^17^. Tomography is important in this distinction and can be essential to rule out possible complications49. However, necrosis can usually only be radiologically detected three to four days after the onset of symptoms^47^ ^,^ ^49^. Thus, the test will only be properly indicated upon admission when there is diagnostic uncertainty, as it may underestimate or incorrectly classify the severity of the disease if obtained less than 72 hours after the onset of symptoms^9^.

In all studies, the etiological suspicion was due to exclusion of the main predisposing risk factors.

Upon admission, it is necessary to collect information about personal or family history of pancreatitis, infectious diseases, known presence of gallstones, consumption of alcohol and medication, and previous surgeries and procedures^47^. The laboratory routine should include liver enzymes, total bilirubin and fractions, serum triglycerides, and calcium^47^. When available and suspected, serological testing for known viruses, as performed in some places^26^ ^,^ ^28^ ^,^ ^30^ ^,^ ^35^, and IgG4 levels for screening for autoimmune pancreatitis^28^ ^,^ ^29^ ^,^ ^31^. Abdominal ultrasonography should be performed in all patients to determine the presence of gallstones and to assess the biliary tract. It is an available, low-cost test, and without exposure to radiation^49^.

Elevated CRP levels are known predictors of severe acute pancreatitis and may be even higher in the presence of COVID-19, their evaluation on admission and during hospitalization being expected^9^ ^,^ ^12^ ^,^ ^49^.

Initial management of acute pancreatitis is supportive and includes close monitoring of vital signs, intravenous fluid resuscitation, pain relief, and adequate nutrition^47^ ^,^ ^49^.

Prophylactic antibiotics are not recommended^49^ and should only be administered as a treatment for confirmed or clinically suspected secondary infection^47^. Most of the included studies mentioned that their patients received empirical antibiotic therapy. Nevertheless, there is no statistically significant reduction in mortality (p=0.07) nor an important reduction in the rates of pancreatic necrosis infection (p=0.47) with their use49.

Local complications should be suspected in the persistence or recurrence of abdominal pain, increased activity of pancreatic enzymes, organ dysfunction, and/or development of signs of sepsis^17^. Local complications of pancreatitis can be acute pancreatic/peripancreatic fluid collections, pancreatic pseudocyst, necrosis, gastric outlet dysfunction, splenic and portal vein thrombosis, and pseudoaneurysms^17^ ^,^ ^47^. Generally, these liquid collections resolve spontaneously and the indication for intervention in pseudocysts is determined by the presence of symptoms^47^ ^,^ ^49^, as happened in one of the reports8. Bleeding from a pseudoaneurysm usually requires an interventional radiologist^47^, something that could not be done in time in the patient reported by Mitrovic et al.^32^. Most cases with sterile necrosis can be treated conservatively, as they also resolve spontaneously over time. Intervention should be considered in the presence of persistent symptoms, for example, and in cases of secondary infection^47^.

In the long term, patients are at risk of developing recurrent episodes of acute pancreatitis, progressing to chronic pancreatitis, and developing endocrine and exocrine insufficiency^9^.

The most serious and commonly described complications of COVID-19, in turn, are inflammation similar to sepsis, coagulopathy, and respiratory or cardiovascular complications^48^. Akarsu et al. showed a correlation between the severity of COVID-19 and pancreatitis, the rate of pancreatitis increasing as the severity of pneumonia increased (p<0.0001). In addition, hospitalization and mortality rates were higher in patients with COVID-19 accompanied by acute pancreatitis43. Similarly, Pandanaboyana et al. found that patients with acute pancreatitis and co-existing SARS-CoV-2 infection have an increased risk of severe acute pancreatitis, worse clinical outcomes, prolonged hospitalization time, and significantly higher 30-day mortality^15^.

Gubatan et al. found that patients with pancreatitis who were also positive for COVID-19 were more likely to require mechanical ventilation compared with patients with pancreatitis without COVID-19^46^. The need for ventilatory support measures was also described among the case reports^18,19,22,23,25,26,28,31,33 37,39,41^.

Coexistence of infection by SARS-CoV-2 and acute pancreatitis can go beyond the probable causality, feeding each other back regarding organ dysfunction. Pancreatitis itself is capable of triggering increased acute lung damage^43^. The hyperinflammatory state of COVID-19 induces indirect, systemic, immune-mediated responses that affect the pancreas^12^, worsening the clinical course of pancreatitis and making it challenging.

Thus, although there are still no robust data that vehemently support SARS-CoV-2 as a precipitating factor for pancreatitis, the evidence accumulated so far has favored this hypothesis. Health teams must be aware of the gastrointestinal repercussions of this virus, since acute pancreatitis can change from a mild and self-limiting illness, which primarily requires supportive measures, to a serious one, with life-threatening complications. 

### Applicability

The results obtained from this review are intended to warn the medical community about the probable pancreatic involvement resulting from SARS-CoV-2, adding evidence about its systemic and not just pulmonary nature. We were careful to select only studies with a diagnosis of acute pancreatitis according to the revised Atlanta Classification, and not only with changes in pancreatic enzymes. In addition, we excluded reports whose patients had some of the main known risk factors. This makes our results even more reliable. 

### Limitations

This review has numerous limitations. Due to the nature of the study, it is not possible to establish a causal relationship between COVID-19 and the onset of acute pancreatitis. We did not conduct a systematic review and, although the integrative review allows the inclusion of studies of different natures16, this opens the way to methodological and statistical heterogeneity. Most publications are still restricted to case reports and, unfortunately, the amount and quality of data provided about medical history and laboratory and radiological investigations varied among them.

Therefore, it is necessary that future studies be conducted to investigate in a more detailed way the associated pathophysiological mechanism. Attention should also be paid to the onset of GI symptoms and their timing in relation to COVID-19 testing, as well as to more common laboratory and imaging findings. We recommend a more comprehensive investigation of the predisposing risk factors during the care of these patients and a better detailing of each case in future publications.

## CONCLUSION

The results from this review corroborate the hypothesis that SARS-CoV-2 infection may be responsible for acute pancreatitis in certain patients, provided that other known etiologies are ruled out. Pathophysiological justifications include pancreatic injury due to the direct cytopathic effect of the virus and due to an immune-mediated response at the expense of the hyperinflammatory state of pneumonia. However, more robust data, capable of establishing a causal relationship between both, should be left to future multicentric studies.
